# Molecular Ultrasound Monitoring of Early Artery Injury After Carotid Balloon Angioplasty

**DOI:** 10.3389/fphar.2018.01569

**Published:** 2019-01-25

**Authors:** Xinhai Mo, Fei Yan, Bo Zhang

**Affiliations:** ^1^Department of Ultrasound in Medicine, Shanghai East Hospital, Tongji University School of Medicine, Shanghai, China; ^2^Paul C. Lauterbur Research Center for Biomedical Imaging, Institute of Biomedical and Health Engineering, Shenzhen Institutes of Advanced Technology, Chinese Academy of Sciences, Shenzhen, China

**Keywords:** ultrasound molecular imaging, collagen IV, artery injury, restenosis, targeted microbubbles

## Abstract

Cardiovascular intervention is a common treatment procedure for many cardiovascular diseases. But restenosis often occurs after these procedures, greatly discounting their long-term therapeutic effects. Early detection of endothelial denudation is helpful for the diagnosis and prevention of restenosis. Here, we fabricated targeted microbubbles by conjugating anti-collagen IV antibodies to the surface of biotinylated microbubbles (MB_ColIV_) and applied them for ultrasound molecular imaging of endothelial injury at early stage. Our results showed that the MB_ColIV_, with a typical multi-peak particle distribution, was successfully constructed, which was confirmed by Alexa Fluor® 555-labeled secondary antibody. *Ex vivo* adhesion of microbubbles revealed that MB_ColIV_ can effectively and specially bind to the surface of balloon-injured carotid artery. The *in vivo* animal experiments showed ultrasound molecular imaging signals from carotid artery-injured rats administrated with MB_ColIV_ were significantly higher than those administrated with isotype control microbubbles. Histological staining of the left carotid common artery revealed that collagen IV was obviously exposed after endothelium denudation in balloon-injured artery. In conclusion, our current study provides an effective approach to detect vascular injury at the early stage and a potential platform for image-guided therapy to vascular injury.

## Introduction

Cardiovascular diseases from atherosclerosis are responsible for the major cause of mortality worldwide (Murray et al., 2012). Pathologically, atherosclerosis is a chronic and progressive disease featured with retention of lipids, accumulation of extracellular matrix and infiltration of smooth muscle cells (SMCs) with or without macrophages (Otsuka et al., 2016). Due to atherosclerotic lesions, cardiovascular events often occur in a complex and unpredictable manner, resulting in a series of complications. Among these, stenosis and thrombosis are the two major complications. Stenosis may cause distal organ ischemia and thrombosis, and trigger thrombotic occlusion of artery to organs, ultimately resulting in life-threatening cardiovascular events.

Early invasive therapies, such as percutaneous coronary intervention (PCI), could improve long-term survival and reduce late myocardial infarction and recurrent unstable angina requiring rehospitalization for patients with non-ST-elevation myocardial infarction (Bavry et al., 2006; Amsterdam et al., 2014). Although the narrowed or obstructed arteries are recovered through endovascular procedures, post-operational restenosis is generally inevitable. The main reason is that mechanical endovascular procedures often cause endothelial damage by activating a series of inflammatory pathways. Due to these inflammatory effects, excessive migration, and proliferation of SMCs, and plentiful synthesis and deposition of extracellular matrix lead to intimal hyperplasia. The serious intimal hyperplasia will result in restenosis which may represent a fatal threat for patients. Evidence demonstrated that restenosis occurs in up to 60% post-angioplasty patients within the first year because of mechanical vascular injury (Petrasheskaya et al., 2016). Therefore, early detection at the stage of endothelial denudation is desirable for the diagnosis and prevention of restenosis.

During vascular injury, many cell adhesion molecules are overexpressed or upregulated. Selectins, integrins, and the immunoglobulin superfamily of cell adhesion molecules are the three major classes of leukocyte cell adhesion molecules (Davis et al., 2003). Numerous studies have shown that intracellular adhesion molecules-1 (ICAM-1), vascular cell adhesion molecules-1 (VCAM-1) or P-selectin can serve as the targets for various imaging probes (Wu et al., 2011; Yan et al., 2018). However, these cell adhesion molecules are not expressed promptly during vascular injury. Generally, it takes 1 day, or even longer, to increase their expression on the surface of vascular endothelial cells (Tanaka et al., 1993; Kennedy et al., 2000).

Collagen IV, representing 50% of the vascular basement membrane, could be exposed immediately when the intact endothelial monolayer is denuded by mechanical injury of vascular intervention (Kalluri, 2003). That is, the expression of collagen IV is time-independent and related to endothelial denudation and damage. Therefore, collagen IV can be used as targeting receptor for detection, and even treatment at the site of vascular injury (Chan et al., 2011; Meyers et al., 2017).

**Figure 1:**
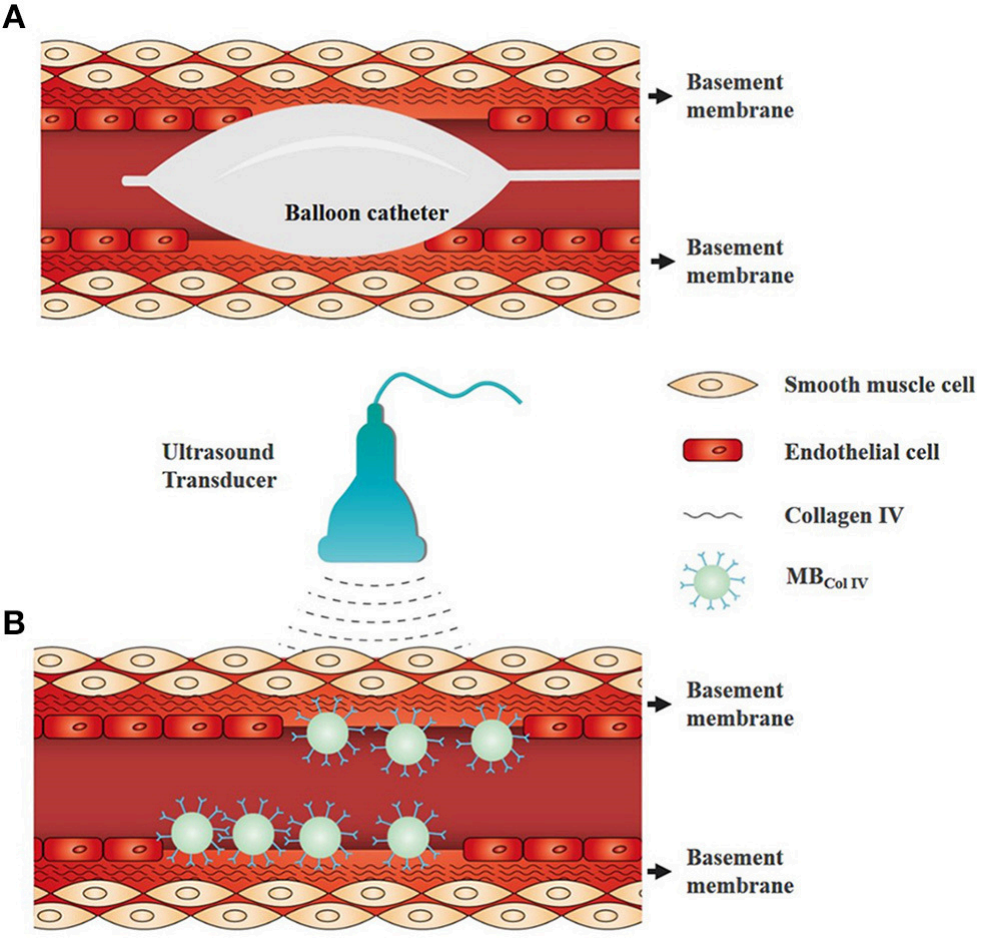
Schematic representation of ultrasound molecular imaging for detecting the vascular injury of endothelium denudation. **(A)** Schematic diagram of balloon angioplasty for endothelial denudation to the carotid artery. **(B)** Schematic of ultrasound molecular imaging for detecting the site of endothelium denudation. Collagen IV is a major component of vascular basement membrane and exposed after endothelium denudation. Collagen IV-targeted microbubbles (MB_ColIV_) were adhered to the vascular injury site without endothelium coverage.

Ultrasonography, a non-invasive and real-time imaging method, is widely used to examine cardiovascular diseases (Yan et al., 2018). With the help of targeted microbubbles (MBs), ultrasound molecular imaging allows to detect specific lesions at molecular level. Generally, the applications of ultrasound molecular imaging require the effective binding of ultrasonic probes to the receptors in the physiologic flow conditions after their intravenous administration. Antibodies, antibody fragments, peptides, and carbohydrates have been conjugated to the surface of MBs. By detecting the signals derived from retained MBs in the target regions, ultrasound molecular imaging can visualize molecular dynamics *in situ*. Currently, several stuieds have been published to demonstrate the diagnostic utility of ultrasound molecular imaging for the detection of inflammation, atherosclerosis and tumor angiogenesis (Villanueva and Wagner, 2008; Willmann et al., 2008; Leong-Poi, 2009; Lindner, 2009; Deshpande et al., 2010). In this study, we fabricated a novel targeted MBs by conjugating anti-collagen IV antibodies to the surface of biotinylated MBs (MB_ColIV_) and applied them for ultrasound molecular imaging of endothelial injury at early stage (Figure 1).

## Materials and Methods

### Preparation of MBs

Biotinylated, lipid-shelled MBs were prepared as previously described (Yan et al., 2013). Briefly, DSPC, DSPE–PEG2000 and DSPE–PEG2000-Biotin (Avanti Polar Lipids, Alabaster, AL, USA) (molar ratios = 9:0.5:0.5) were blended in chloroform. The solvent was evaporated under nitrogen flow at room temperature, forming a thin film on the wall of the test tube. Residual chloroform was further removed by vacuum treatment for at least 2 h. The completely dried phospholipid membrane was hydrated at 60°C with a given buffer consisting of 0.1 M Tris (pH 7.4):glycerol:propylene glycol (80:10:10 by volume), and then transferred into vials. After sealing the vials, the air in the vials was replaced with perfluoropropane (C_3_F_8_). Biotinylated MBs (MB_biotin_) were obtained by mechanically vibrating for 30 s. The resulting MB_biotin_ were washed with phosphate buffered saline (PBS) solution twice to eliminate excess unincorporated lipids by centrifuge at 400 g for 3 min. Three microgram of avidin per 10^7^ MBs was then incubated with the MB_biotin_ dispersion for 30 min at room temperature, followed by rinsing three times to remove unreacted avidin (Aladdin, Pudong, Shanghai, China). Then either 2.5 μg of biotinylated goat anti-rat Type IV Collagen antibody or biotinylated goat IgG isotype control antibody (SouthernBiotech, Birmingham, AL, USA) was added to per 10^8^ MBs. After incubation for 30 min at room temperature, MB_ColIV_ or isotype control MBs (MB_Ctrl_) were collected by centrifugation. Non-targeted MBs (MB_N_) without conjugated primary antibody were also prepared.

### Characterization of MBs

To confirm the conjugation of Type IV Collagen antibody to the surface of MBs, Alexa Fluor® 555-labeled anti-goat secondary antibody (Invitrogen, Carlsbad, CA, USA) was added to MB_ColIV_ and mixed for 30 min at room temperature. The mixture was separated by centrifugation and the upper layer was washed twice with PBS to eliminate the free secondary antibody. The fluorescence-labeled MB_ColIV_ were examined by a fluorescence inversion microscopy (Olympus Corporation, Tokyo, Japan).

To analyze the characteristics of the MB_ColIV_, MB_N_ were used as the blank control. The morphology of the MBs were examined by using bright-field and fluorescence inversion microscopy (Olympus Corporation, Tokyo, Japan). Particle size, size distribution and concentration of MBs were determined by using the Accusizer 780 Optical Particle Sizer (Particle Sizing Systems, Santa Barbara, CA, USA) with a 0.5 μm diameter detection limit.

### Carotid Artery Injury Model

All animal procedures were performed in accordance with the principles outlined in the Guide for the Care and Use of Laboratory Animals and approved by Animal Care and Use Committee. Adult male Sprague–Dawley rats (Vital River Laboratory, Beijing, China), weighing 400–500 g, underwent the rat carotid artery balloon injury as previously described (Chan et al., 2011; Petrasheskaya et al., 2016). Rats were given aspirin by oral gavage before surgery and kept anesthetized by isoflurane at 2% at 2 L/min oxygen. Following a midline neck incision, the left common carotid artery (LCCA), left external carotid artery (LECA), and left internal carotid artery (LICA) were exposed. After distal ligation of the LECA, the proximal end of LCCA and the distal end of LICA were then temporarily clipped by arterial clamps and a 2-French arterial embolectomy catheter (Edwards Lifesciences, Irvine, CA, USA) was advanced proximally into LCCA through the incision of LECA. The balloon was inflated and withdrawn with a rotating action to the arteriotomy in LECA and then reintroduced and retracted twice again. The balloon catheter was removed and the LECA was ligated. Finally, arterial clamps were removed to restore the blood flow, and then the neck incision was closed. Thereafter, rat carotid artery injury model was performed and used for the following experiments.

### Histological Analysis

Bilateral carotid arteries were harvested following *in situ* perfusion fixation with PBS and 4% paraformaldehyde. Tissues were placed in paraformaldehyde for 1 h at 4°C, then overnight in 30% sucrose in PBS at 4°C for cryo-protection. Vessels were coated with Optimum Cutting Temperature O.C.T.™ compound (Tissue Tek, Hatfield, PA) and transferred to liquid nitrogen for flash-freeze. Vessels were sectioned to obtain arterial cross-sections across the length of the artery by cryostat microtome (CM1950; Leica, Heidelberg, Germany) and were examined histologically using routine hematoxylin-eosin (H&E) staining for morphometric analysis. Digital images were collected with bright-field by inversion microscopy (Olympus Corporation, Tokyo, Japan).

### *Ex vivo* Adhesion of MBs

Attachment ability of MBs to collagen IV was evaluated by counting the number of adhering MBs. Briefly, after the rat carotid artery injury model and the *in situ* perfusion fixation were completed, the LCCA was harvested and then opened longitudinally. The side of adventitia was adhered to a coverslip. Then MBs were added into a 6-well plate (2 × 10^7^ MBs per well) and filled with PBS. Due to the static flotation nature of MBs, the coverslip with the artery coated faced downward to maximize interaction between the tissue and MBs for 5 min at room temperature. After that, the free MBs were removed by rinsing five times with PBS. To further determine the specificity of MBs adhesion, vessel tissues were pretreated with 25 μg/mL goat anti-rat Type IV Collagen antibody to block available receptors prior to MB_ColIV_ incubation. Digital images were obtained with bright-field by inversion microscopy (Olympus Corporation, Tokyo, Japan) to count the number of attached MBs in five random fields of view.

### *In vivo* Ultrasound Molecular Imaging

After rat carotid artery injury model was developed as described above, rats were kept anesthetized on a heated stage throughout the imaging session. Ultrasound molecular imaging was performed with a commercial ultrasound system Resona 7 (Mindray Medical Systems, Shenzhen, China) using a L11-3 linear array transducer. Contrast imaging mode was applied in the ultrasound molecular imaging experiments. All imaging parameters were kept constant throughout the whole procedure as follows: frequency 5.6 MHz, depth 2 cm, gain 45 dB, frame rate 10 Hz, dynamic range 115 dB, and mechanical index 0.085. In order to reduce motion interference, both the transducer and the rats were fixed to maintain the same long axis cross section of carotid artery. The concentration of MBs suspension was adjusted to 2 × 10^8^ MBs/ml. Then 200 μl MBs suspension was injected intravenously through tail vein following by flushing with 50 μl PBS. Four minutes after MBs injection, 100 frames of images were captured to obtain a signal from adherent and freely circulating MBs. A continuous high-power destructive pulse (mechanical index: 0.553) was then applied for 2 s to destroy these MBs. After 2 s, to allow the freely circulating MBs to replenish, another 100 frames of images were acquired, in which the ultrasonic signals were from any residual freely circulating MBs and tissue. To minimize the bias and test the specificity of these molecular imaging signals only resulting from adherent targeted MBs, MB_ColIV_, and MB_Ctrl_ were administered in random order to all rats. Another 30-min delay was allowed to clear MBs from the preceding imaging session. As a control, ultrasound molecular imaging was also performed in normal rats to further assess the specificity of MBs adhesion. The acoustic imaging signals were analyzed by using commercially available analysis software (Mindray Medical Systems, Shenzhen, China). As previously described (Wu et al., 2011), the difference in signal intensity from adherent MBs was calculated by subtracting the post-destruction signal from the pre-destruction signal.

### Immunohistochemistry

Immunostaining analysis for collagen IV was performed to confirm that collagen IV was exposed through endothelium denudation. Briefly, LCCAs from normal artery group and balloon-injured artery group were excised, followed by *in situ* perfusion fixation with PBS and 4% paraformaldehyde. Then artery samples were transferred to paraformaldehyde overnight, followed by ethanol dehydration and paraffin embedment. Artery samples were sectioned to obtain arterial cross-sections. After the process of deparaffinage and rehydration, slides were blocked and then incubated with the primary antibody goat anti-rat Type IV Collagen (SouthernBiotech, Birmingham, AL, USA), followed by being rinsed with PBS thrice and then incubated with horseradish peroxidase-conjugated secondary antibodies. Finally, slides were incubated with diaminobenzidine for horseradish peroxidase reaction. In order to exhibit the tissue morphology of vascular wall, slides were counterstained with hematoxylin. Immunohistochemical images were acquired by using inversion microscopy (Olympus Corporation, Tokyo, Japan) under bright field.

### Statistical Analysis

Data were analyzed by the statistic software SPSS 20.0 (IBM Corp, Armonk, New York, USA). As values were normally distributed, statistic difference between two groups was determined by Student's *t*-test, and one-way ANOVA were used to compare among more than two groups. If the differences among these groups were significant for one-way ANOVA, the difference between the two groups was conducted by Student-Newman-Keuls test. The *P* < 0.05 indicated statistically significant difference.

## Results

### Characteristics of MBs

**Figure 2:**
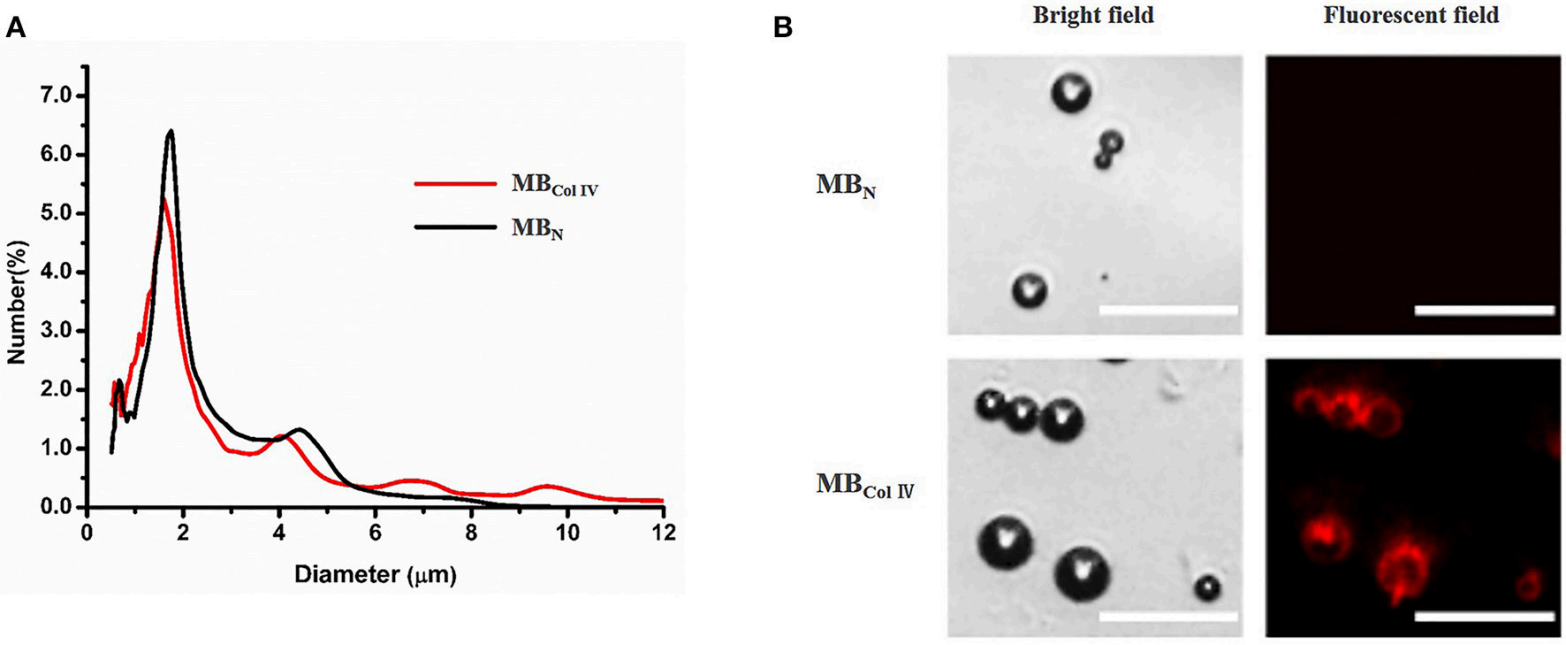
Characterization of ultrasound contrast agents. **(A)** Size distribution of non-targeted microbubbles (MB_N_) and Collagen IV-targeted microbubbles (MB_ColIV_). The size distribution curves of MB_N_ and MB_ColIV_ were similar. **(B)** MB_N_ and MB_ColIV_ under bright–field and fluorescence microscopy. The red fluorescence indicated the conjugation of Alexa Fluor® 555-labeled secondary antibody with the Type IV Collagen antibody (Scale bar = 15 μm).

The fabricated MB_ColIV_ appeared a typical multi-peak particle distribution. There was no significant difference between MB_ColIV_ and MB_N_ for the particle size and distribution (all *P* > 0.05). The mean diameter of MB_ColIV_ and MB_N_ were 1.92 and 1.89 μm, respectively. The results demonstrated that the conjunction of avidin and biotinylated antibody to MBs surface did not change the particle size and distribution of MBs (Figure 2A). Figure 2B showed that both MB_ColIV_ and MB_N_ had smooth and spherical morphology for microscopic observation. The surface of MB_ColIV_ emitted a bright red fluorescence, indicating the anti-rat Type IV Collagen antibodies were successfully conjugated onto the surface of these MBs (Figure 2B).

### Confirmation of Rat Carotid Artery Injury

**Figure 3:**
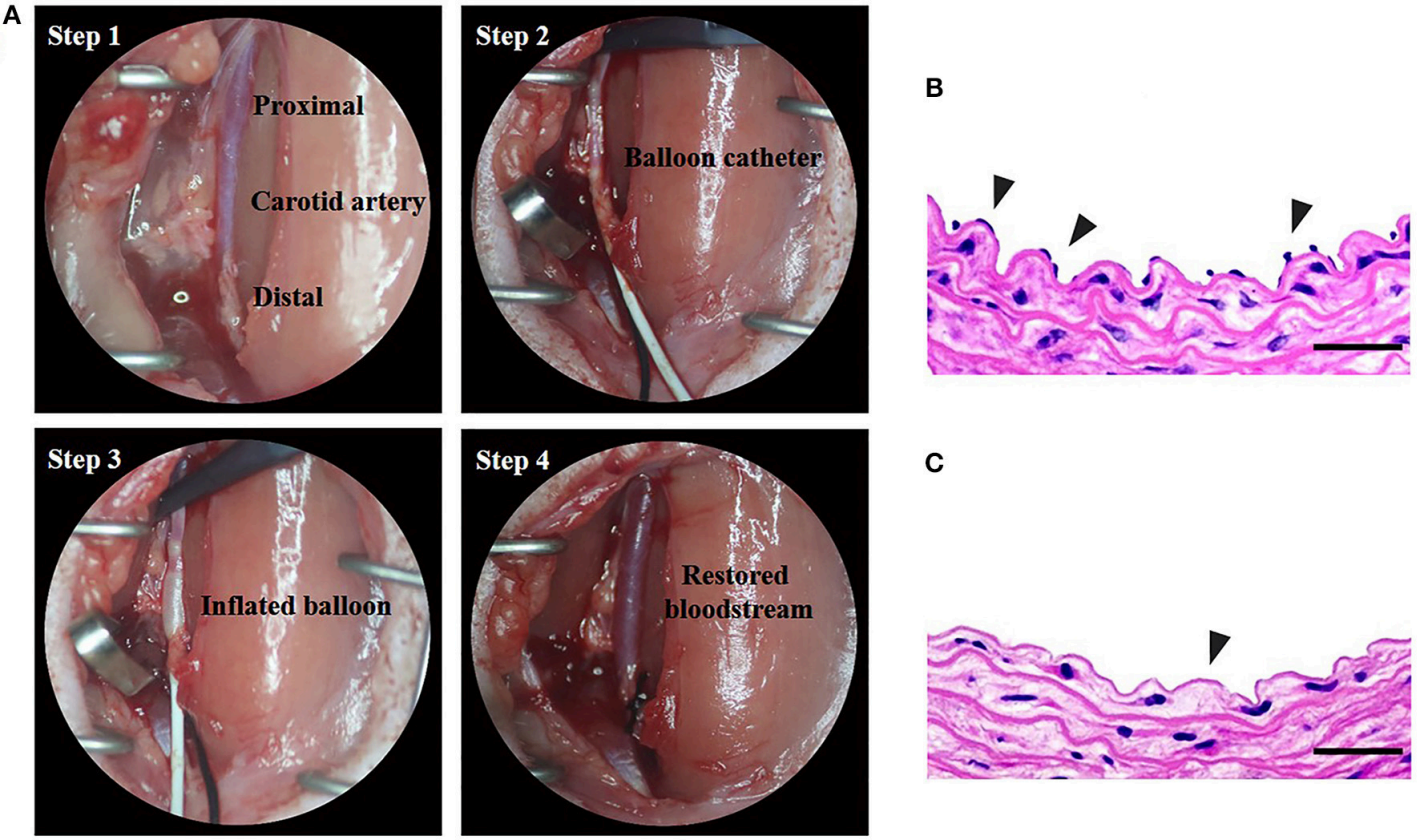
Construction of rat carotid artery injury model. [**(A)** Step 1] The left carotid common artery (LCCA) has been exposed. [**(A)** Step 2] the proximal LCCA and the distal left internal carotid artery (LICA) have been temporarily clipped by arterial clamps. 2-French uninflated balloon catheter has been inserted into the LCCA through the incision of the left external carotid artery (LECA). [**(A)** Step 3] The balloon has been inflated and will be withdrawn with a rotating action to the incision of LECA. The procedure of reintroduction and retraction will be repeated thrice. [**(A)** Step 4] Remove the catheter and ligate LECA, and then remove arterial clamps to restore the blood flow. **(B,C)** The H&E staining of normal and balloon-injured carotid artery. **(B)** Normal right carotid artery. Note the endothelial monolayer (arrowheads) lines the lumen of artery underlying the media. **(C)** Balloon-injured left carotid artery. The left carotid artery which was harvested immediately after balloon injury procedure exhibits endothelial denudation in the intima (arrowheads) (Scale bar = 150 μm).

Vascular balloon injury is a standardized protocol for endothelial denudation and intimal damage through repeated insertion and retreat of the balloon catheter (Figure 3A). In order to confirm the successful development of carotid artery injury model, cross-sections were stained with H&E. From the Figures 3B,C we can see that LCCA lost an endothelial monolayer after vascular balloon injury, compared with the normal right common carotid artery (RCCA). The results showed that we successfully constructed the carotid injury model.

### Binding Specificity of MBs *ex vivo*

**Figure 4:**
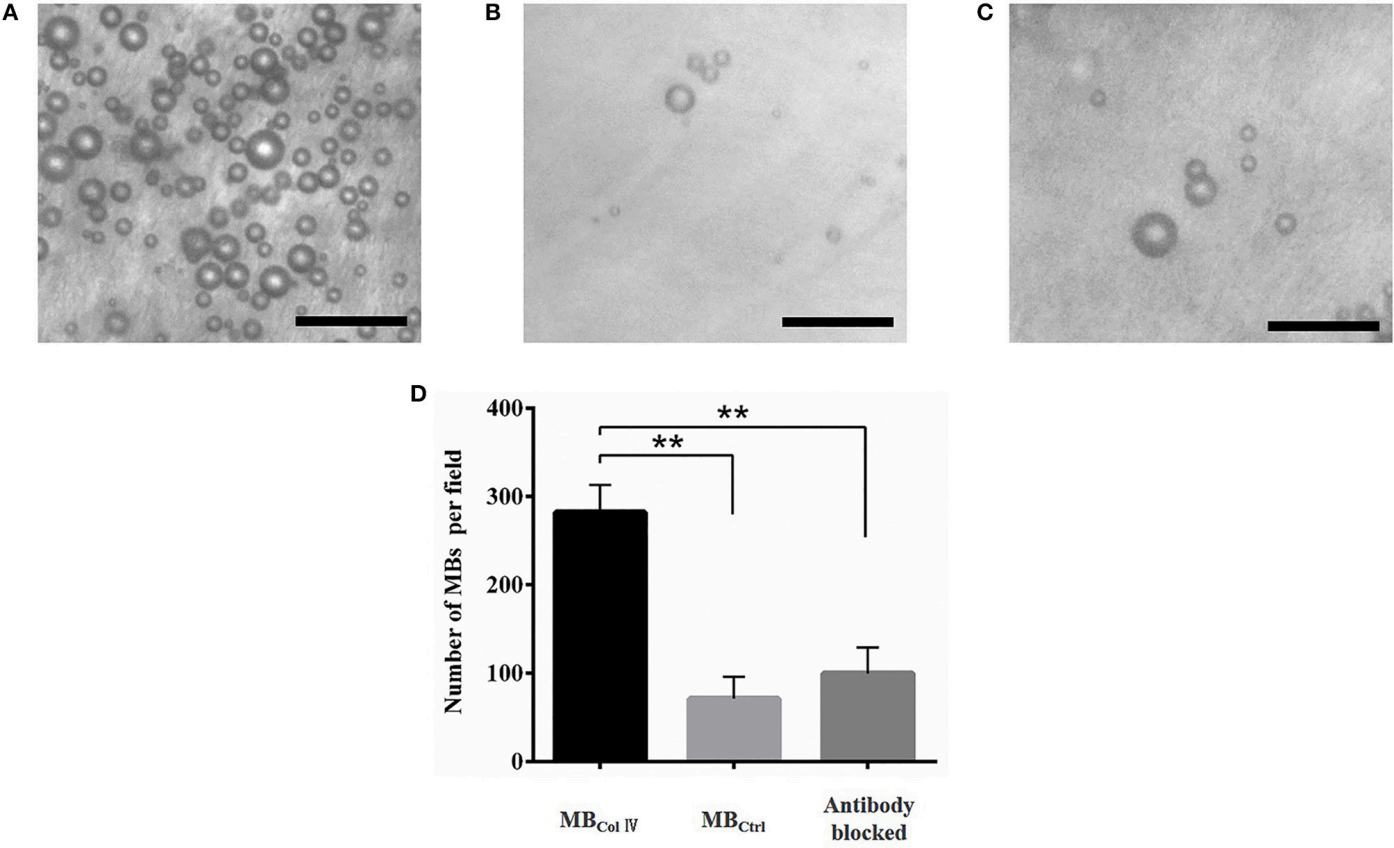
*Ex vivo* adhesion of MBs to the balloon-injured carotid artery. Representative bright-field micrographs show the balloon-injured carotid artery after incubation with MB_ColIV_ **(A)**, MB_Ctrl_ **(B)**, or MB_ColIV_ with antibody blocking **(C)**. MB_ColIV_ adhesion is significantly higher than MB_Ctrl_. But there is significantly lower adhesion of MB_ColIV_ after blocking the receptors with the Type IV Collagen antibody. (Scale bar = 20 μm). **(D)** Quantitative assay for MBs adhesion to the balloon-injured carotid artery ^**^P < 0.01 (*n* = 3).

To determine the binding specificity of MB_ColIV_, we performed the binding specificity experiments *ex vivo* through incubating the MB_ColIV_ or MB_Ctrl_ with the injured LCCA. After a static exposure of the injured LCCA to MBs, we could see that a large number of MB_ColIV_ were adhered to the injured vessel (Figure 4A), which was significantly higher than MB_Ctrl_ (Figure 4B). Meanwhile, pre-blocking receptors in injured LCCA by anti-rat Type IV Collagen antibodies greatly reduced the attachment of MB_ColIV_ (Figure 4C). Quantitative analysis indicated that the binding efficiency of MB_ColIV_ was about four times higher compared with MB_Ctrl_ (282.1 ± 77.2 MBs per field vs. 71.07 ± 62.07 per field, *P* < 0.01) (Figure 4D).

### Ultrasound Molecular Imaging

**Figure 5:**
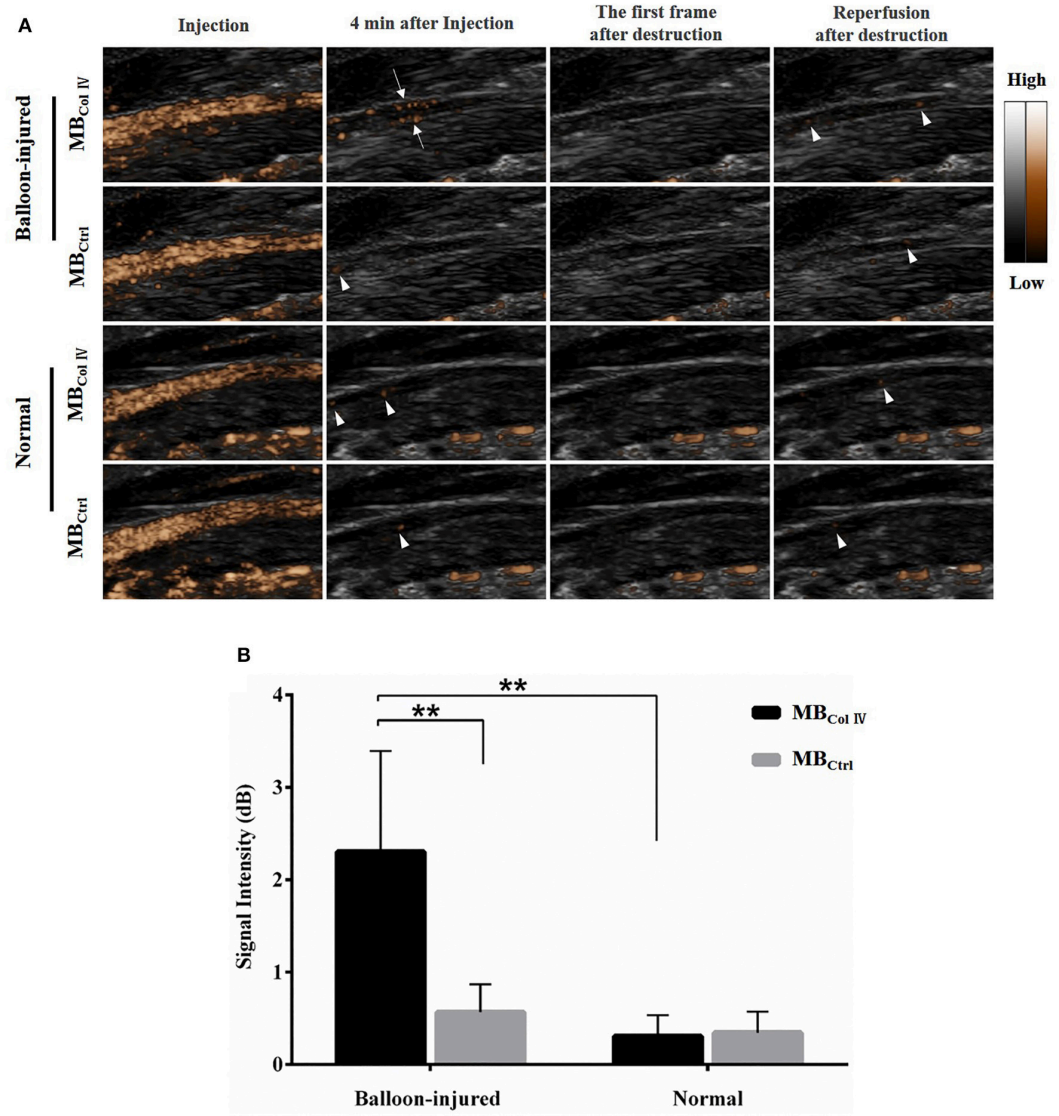
Ultrasound molecular imaging of balloon-injured (*n* = 8) and normal (*n* = 7) carotid arteries and the quantitative analysis of signal intensity. **(A)** Representative results of ultrasound molecular imaging show the long-axis images of rat carotid arteries. MB_ColIV_ are obviously bound to the wall of balloon-injured vascular (arrows) but hardly to normal vascular. And ultrasound imaging also exhibits MB_Ctrl_ freely circulate in either balloon-injured vascular or normal vascular (arrowheads). **(B)** The quantitative analysis of signal intensity in balloon-injured (*n* = 8) and normal (*n* = 7) carotid artery after bolus injection of MB_ColIV_ or MB_Ctrl_. ^**^P < 0.01 for MB_ColIV_ vs. MB_Ctrl_ in balloon-injured group, and for MB_ColIV_ in balloon-injured group vs. normal group.

To further evaluate the special adherence of MBs and their ultrasound imaging effect *in vivo*, ultrasound molecular imaging was performed in the balloon-injured carotid artery group (*n* = 8) and the normal carotid artery group (*n* = 7). As shown in Figure 5A, 4 min after MBs injection, almost all of MBs had been washed out. Meanwhile, MB_ColIV_ were hardly attached to the normal artery wall (Figure 5A). By contrast, MB_ColIV_ were obviously adhered to the inner wall of balloon-injured artery (Figure 5A and Supplementary Video 1). After the trigger of the destruction pulse, no echo signal of MBs was found in balloon-injured the carotid artery group and the normal carotid artery group (Figure 5A). This procedure further confirmed that MB_ColIV_ were attached to the inner vessel wall. Quantitative analysis revealed that the signal intensity of MB_ColIV_ (2.30 ± 0.91 dB) was higher than MB_Ctrl_ (0.57 ± 0.26 dB) in balloon-injured carotid artery group (*P* < 0.01) (Figure 5B). Moreover, the signal intensity of MB_ColIV_ in the balloon-injured carotid artery group was also significantly higher than normal carotid artery group (2.30 ± 0.91 dB vs. 0.31 ± 0.21 dB, *P* < 0.01) (Figure 5B).

### Immunohistochemistry

**Figure 6:**
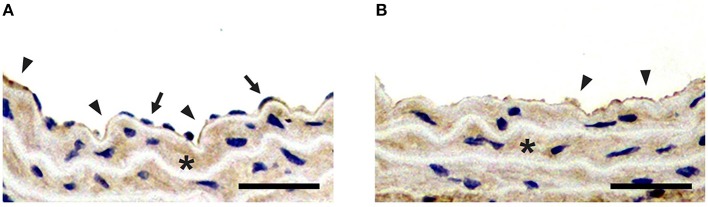
Immunohistochemical staining for collagen IV. **(A)** Immunohistochemical staining of the left carotid common artery (LCCA) from normal group. **(B)** Immunohistochemical staining of LCCA from balloon-injured group. Positive staining for collagen IV is presented in both normal and balloon-injured artery (brown stain; arrowheads). Compared with the endothelium (arrows) coverage for collagen IV (arrowheads) in normal artery, collagen IV is exposed after endothelium denudation in balloon-injured artery (arrowheads). Besides, Collagen IV is also localized to tunica media (asterisk) (Scale bar = 150 μm).

To confirm the results of ultrasound molecular imaging, LCCAs of the normal group and balloon-injured group were harvested and analyzed for the exposure of collagen IV after endothelium denudation. The results of immunohistochemical staining examination were presented in Figure 6. We could see that both balloon-injured artery and normal artery were stained dark brown for collagen IV in basement membrane, indicating collagen IV was a major constituent of the basement membrane. It was consistent with the previous studies (Kalluri, 2003; Abreu-Velez and Howard, 2012; Manon-Jensen et al., 2016). In normal artery intima, collagen IV presenting in basement membrane, was covered by a monolayer endothelium (Figure 6A). However, without endothelium coverage, collagen IV was directly exposed in balloon-injured artery (Figure 6B). The exposure of collagen IV greatly enhanced the interaction opportunity with MB_ColIV_. In addition, collagen IV was also a component of the basement membrane for SMCs in tunica media, which had been reported in previous studies (Shekhonin et al., 1985; Howard and Macarak, 1989).

## Discussion

Microbubble-based ultrasound molecular imaging is a promising approach in the disease diagnosis at the early stage. This is especially the case for vascular disorders since the microscale-targeted bubbles only exist in the blood and are not extravasate from the circulation. This feature makes it greatly standing out from other nanoparticle-based molecular imaging modalities, such as quantum dot-based optical molecular imaging and iron nanoparticle-based magnetic imaging resonance (Lobatto et al., 2011). Moreover, ultrasound is a safe, affordable and real-time imaging modality and can detect deep-seated diseased tissues. All of these features of ultrasound prompted us to explore the diagnosis feasibility of injured artery by using ultrasound molecular imaging.

In our study, we fabricated the targeted MB_ColIV_ by biotin-avidin bridging method. As shown in Figure 2B, collagen IV antibodies were successfully conjugated to the surface of MBs. It has to be pointed out that we did not use the *in vitro* tool in our static binding experiments such as culture plates, pre-coated with Matrigel, which has been taken to examine the binding of targeted particles to collagen IV (Dong et al., 2016). In fact, they are not suitable in our experiments. Due to the Matrigel's water solubility, Matrigel dissolves in PBS during rinsing, and almost all MBs would be washed away (data not shown). Therefore, we used the harvested tissue of injured carotid artery to examine the affinity effect of MBs. Effective and special targeting affinity was achieved in our *ex vivo* binding experiments (Figure 4). The number of MB_ColIV_ binding to the tissue of injured artery was nearly four times higher than MB_Ctrl_, indicating MB_ColIV_ we obtained can be used as a potential ultrasound molecular imaging probe.

In our present study, we used intravenously injection for ultrasound molecular imaging *in vivo* in order to determine whether MB_ColIV_ could adhere to the site of endothelial denudation and be visually detected by a clinical ultrasound scanner or not. Surprisingly, we found stronger ultrasound molecular imaging signals from injured vascular wall after MB_ColIV_ injection but not MB_Ctrl_, the former being nearly five times higher than MB_Ctrl_. Interestingly, no obvious ultrasound molecular imaging signals were detected from the normal arterial wall after MB_ColIV_ or MB_Ctrl_ injection. These results suggested that MB_ColIV_ had a highly specific binding ability to these collagen IV targets and could function as an ultrasound molecular imaging probe to detect vascular injury. To take the drug-loaded advantages of MBs into consideration, it is easy to design the MB_ColIV_ into drug-loaded targeted MBs through carrying drugs (Lammertink et al., 2015), gene (Zhao et al., 2018) and/or especially therapeutic gas, such as nitric oxide, hydrogen sulfide, carbon monoxide, and so on (Fix et al., 2015). If that is the case, we have reasons to believe that MB_ColIV_ could be further functionalized as theranostics agent and would play a great role for ultrasound imaging-guided treatment of vascular injury.

There are several limitations in our study. The rat carotid artery balloon injury model described in our study imitate vascular injury by angioplasty. However, in clinical practice, restenosis occurs in patients who have occlusive atherosclerotic lesions after dilating the narrow lumen through angioplasty. Besides, to evaluate non-culprit stenosis severity, the instantaneous wave-free ratio, which is helpful for guiding PCI strategy, has been regarded as the promising method and used in patients with acute coronary syndrome and multivessel disease (Indolfi et al., 2015). In order to reduce complications, coronary balloon angioplasty is usually combined with stent implantation during PCI (Byrne et al., 2017). Compared with human angioplasty, the rat carotid artery balloon injury model is lacking atherosclerotic lesions and stent implantation. Whereas, the above artery injury model is widely perceived as a well-defined model for researching vascular remodeling and vascular cell proliferation, and it is only suitable for the proof-of-concept study. Another limitation is that once the collagen underlying the basement membrane is exposed, it may cause thrombogenesis, producing an obstruction for targeted MBs. To prevent thrombogenesis, an anticoagulant drug is used before balloon manipulation, but this procedure does not affect the prognosis of restenosis model (Chan et al., 2011; Riegler et al., 2013). Furthermore, the method of avidin-biotin linkage is unlikely to be applied in human clinical practice because of the potential immune response. Therefore, a covalent linkage method is expected to be used to develop targeted contrast agents in the future. For instance, small molecules such as biotin and peptides can be attached to the shell-forming material (protein or lipid) before bubble generation (Weinkauf et al., 2018).

In summary, we have successfully fabricated MB_ColIV_ by conjugating anti-collagen IV antibodies to the surface of MBs and confirmed the feasibility to apply them for ultrasound molecular imaging of endothelial injury at early stage. Our study provides an early diagnosis tool for restenosis due to endothelial damage and a potential platform for image-guided therapy for vascular injury.

## Author Contributions

XM and BZ initialed the project. XM and FY conducted the experiment and data analysis. BZ and FY provided equipment, materials, and experimental methods. XM wrote the manuscript. FY proofread the manuscript. All authors agreed with the final version of the manuscript.

### Conflict of Interest Statement

The authors declare that the research was conducted in the absence of any commercial or financial relationships that could be construed as a potential conflict of interest.
